# A Selective Histamine H4 Receptor Antagonist, JNJ7777120, Role on glutamate Transporter Activity in Chronic Depression

**DOI:** 10.3390/jpm12020246

**Published:** 2022-02-09

**Authors:** Yesim Yeni, Zeynep Cakir, Ahmet Hacimuftuoglu, Ali Taghizadehghalehjoughi, Ufuk Okkay, Sidika Genc, Serkan Yildirim, Yavuz Selim Saglam, Daniela Calina, Aristidis Tsatsakis, Anca Oana Docea

**Affiliations:** 1 Department of Medical Pharmacology, Faculty of Medicine, Ataturk University, Erzurum 25240, Turkey; yesim.yeni@atauni.edu.tr (Y.Y.); ufukokkay@atauni.edu.tr (U.O.); stulay.gnc@gmail.com (S.G.); 2 Department of Emergency Medicine, Faculty of Medicine, Ataturk University, Erzurum 25240, Turkey; 3 Department of Pharmacology and Toxicology, Faculty of Veterinary Medicine, Ataturk University, Erzurum 25240, Turkey; 4 Department of Pathology, Faculty of Veterinary Medicine, Ataturk University, Erzurum 25240, Turkey; syildirim@atauni.edu.tr (S.Y.); yssaglam@atauni.edu.tr (Y.S.S.); 5 Department of Clinical Pharmacy, University of Medicine and Pharmacy of Craiova, 200349 Craiova, Romania; 6 Department of Forensic Sciences and Toxicology, Faculty of Medicine, University of Crete, 71003 Heraklion, Greece; 7 Department of Analytical and Forensic Medical Toxicology, Sechenov University, 119991 Moscow, Russia; 8 Department of Toxicology, University of Medicine and Pharmacy of Craiova, 200349 Craiova, Romania; daoana00@gmail.com

**Keywords:** depression, glutamate, H4, hippocampus, JNJ7777120

## Abstract

Glutamate release and reuptake play a key role in the pathophysiology of depression. glutamatergic nerves in the hippocampus region are modulated by histaminergic afferents. Excessive accumulation of glutamate in the synaptic area causes degeneration of neuron cells. The H4 receptor is defined as the main immune system histamine receptor with a pro-inflammatory role. To understand the role of this receptor, the drug JNJ7777120 was used to reveal the chronic depression-glutamate relationship. We have important findings showing that the H4 antagonist increases the glutamate transporters’ instantaneous activity. In our experiment, it has been shown that blocking the H4 receptor leads to increased neuron cell viability and improvement in behavioral ability due to glutamate. Therefore, JNJ can be used to prevent neurotoxicity, inhibit membrane phospholipase activation and free radical formation, and minimize membrane disruption. In line with our findings, results have been obtained that indicate that JNJ will contribute to the effective prevention and treatment of depression.

## 1. Introduction

Chronic depression is a mental disorder that affects around 5% of the adult population globally and was one of the top 25 leading causes of burden worldwide in 2019 [1] and increased in prevalence during 2020 due to the COVID-19 pandemic [2]. The current situation regarding the increasing prevalence of chronic depression requires health systems to find a solution for promoting mental wellbeing and treating those affected in order to prevent the socioeconomic impact of this disorder. Depression is a mental disorder that has a negative impact both on psychical and physical health, affecting cognition and human relationships and leading to disability and increasing the burden of disease [3]. Tathophysiology of depression continues to be incompletely known and associated with the heterogeneous pattern determined by multiple mechanisms of development and several aetiologies [3]. These ideas are supported by the high number of patients that don’t respond to antidepressant therapy [4]. There are several mechanisms considered responsible for the pathophysiology of depression, such as the involvement of dysregulation in biogenic amine, dysregulation of HPA (hypothalamic-pituitary-adrenal) axis, dysregulation in neurogenesis and second messenger systems, genetic, immunological factors, environmental factors, and the upregulation of CRF (corticotrophin-releasing factor), but none of these can explain all the signs and symptoms observed in these patients [3].

In recent years, many studies have shown that glutamate (glut), an excitatory neurotransmitter, plays a role in the pathophysiology of chronic depression [5,6,7]. Many studies have shown that different stress factors cause an increase in glut concentration in the synaptic gap in the hippocampus region and a change in glut (receptor, transporter (EAAT1, EAAT2), and glutamine synthetase (glul) functions [8,9,10,11,12,13]. It is also known that glutamatergic nerves in the hippocampus region are modulated by histaminergic afferents [14]. But the modulation role of histamine with glut receptors and transporters in chronic depression is not clear.

We have important findings showing that the H4 antagonist increases the glut transporters’ instantaneous activity. However, it has been reported that histamine H1 and H2 receptors modulation cause glut release in astrocytes, while the H3 receptor suppresses glutamatergic neurons [15,16].

The H4 receptor (H4R) is defined as the main immune system histamine receptor with a proinflammatory role [17]. In order to understand the role of this receptor, the drug JNJ7777120 was used to reveal the chronic depression-glut relationship. The present study was to investigate the role of the potent and selective H4R antagonist JNJ7777120 on the activity of glut transporters in a model of chronic unpredictable mild stress. In our study for evaluation of JNJ7777120, we prepared control groups by using Sertraline (routinely used in patients with depression), Valproic acid (medication used for bipolar and epilepsy disorders) and Ceftriaxone (an antibiotic that increases glutamate transporter activity).

Sertraline increases the release of 5-HT by inhibiting the 5-HT transporter. This drug is prescribed because of its efficacy as well as its relatively mild side effects and because it is less toxic in overdose than other anti-depressants. Sertraline is primarily used to treat major depression in adult patients [8,10]. Its neuroprotective and anti-oxidant effects have also been reported in neurodegenerative diseases [12,13].

Valproic acid has been shown to protect neurons from glut-induced excitotoxicity and has anti-inflammatory and anti-oxidant properties [18]. It also demonstrated the ability to reduce immobility time in the forced swimming test, a significant measure of behavioral hopelessness in the depression model, and increased tolerance to stress in the learned helplessness paradigm [19]. However, the antidepressant mechanisms of valproic acid are not fully understood.

Ceftriaxone is a third-generation cephalosporin under the group of β-lactam antibiotics and has neuroprotective effects [20]. Among the different possible mechanisms of ceftriaxone, it is known to be involved in the up-regulation of excitatory amino acid transporter EAAT-2 expression, the attenuation of oxidative stress and neuroinflammation, and to have neuroprotective effects [21].

## 2. Materials and Methods

### 2.1. Chemicals

Ceftriaxone (Unacefin 1g, IV vial, Yavuz İlaç Ecza Deposu Medikal Urunleri San. ve Tic. A.Ş, Istanbul, Turkey), Sertraline (Selectra 100 mg, film tablet, Arven İlaç San ve Tic. A.Ş, Istanbul, Turkey), Valproic acid and JNJ7777120 (Sigma-Aldrich, St. Louis, MO, USA), Probes master mix, high pure RNA isolation and transcriptor first-strand cDNA synthesis kit (Roche, Basel, Switzerland), AChE (Sunlong, Biotech) Co., Ltd., Zhejiang, China), lactate dehydrogenase and glutathione ELISA kit (Elabscience, TX, USA), glutamine synthesis, JWA, EAAT1 and EAAT2 primer-probe mix were obtained from Thermo Fisher (Waltham, MA, USA).

### 2.2. Experiment Animals

Seventy-two male (eight weeks old) Sprague Dawley rats weighing 200–250 g were obtained from Ataturk University Experimental Animal Breeding Unit. This study was carried out with the approval of Atatürk University Experimental Animal Ethics Committee dated 13.08.2018 and numbered 42190979-000-E.1800235611. 

### 2.3. Chronic Unpredictable Mild Stress Model

**Figure 1:**
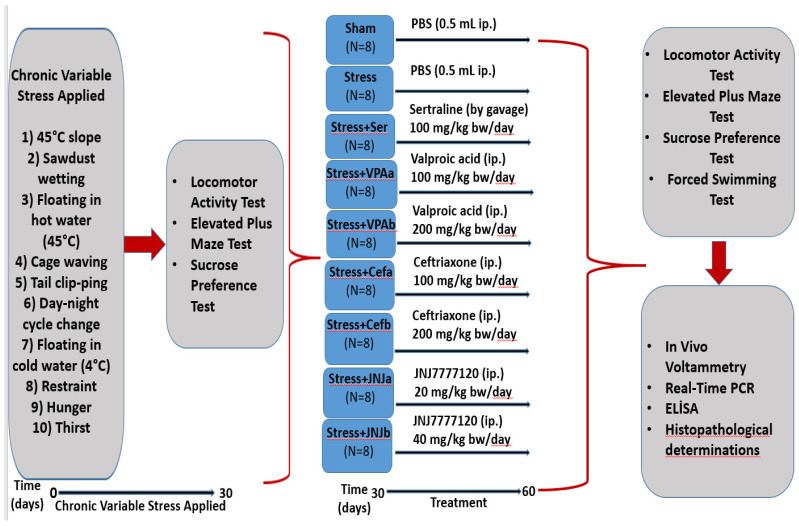
Flow chart of the study. Schedule of the experimental procedures. The experiment has two steps. The mild stress model was established between 0–30 days. The treatment starts from day 30–60. Different analyses were done on the 30th day and the 60th day.

The animals from the mild stress groups (64 rats) were subjected to stressor applications such as 45-degree slope, sawdust wetting, floating in hot water (45 °C), cage waving, tail clipping, day-night cycle change, floating in cold water (4 °C), restraint, hunger, and thirst at different times of the day to minimize the possibility of predicting stress exposure by the rats [22]. Stressors were applied at moderate unpredictable times for 30 days (each stress factor was applied throughout the day). The animals from the control group (eight rats) were kept in standard animal conditions with free access to feed and water (Figure 1).

### 2.4. Treatment

The experimental groups were randomly divided into nine groups with eight animals in each group as follows: Control, Stressed (Stress), Sertraline 100 mg/kg (Ser) (gavage), Valproic acid 100 (VPA_a_) and 200 mg/kg (VPA_b_), Ceftriaxone 100 (Cef_a_) and 200 mg/kg (Cef_b_), JNJ7777120 20 (JNJ_a_) and 40 mg/kg (JNJ_b_) (i.p) (Figure 1).

### 2.5. Locomotor Activity Test

Anxiety-like behavior was measured using a square lattice (50 × 50 × 50 cm, O’Hara and Co., Ltd., Tokyo, Japan) (n = 8). The distance traveled and the time spent by each animal in the central region were recorded for 30 min by using a video imaging system (EthoVisionXT; Noldus Information Technology, Wageningen, The Netherlands) [23].

### 2.6. Elevated plus Maze Test

Anxiety-like behavior was measured using the elevated plus-maze (50 cm long, 14 cm wide, 50 cm high; O’Hara and Co., Ltd., Tokyo, Japan). Each animal was placed in the middle area of the maze facing one of the open arms (n = 8). Time spent in the open arms was measured over 5 min with the EthoVision XT video imaging system [24]. 

### 2.7. Forced Swimming Test

On day 60, the rats were floated one by one in glass cylinders 25 cm high filled with water, with their tails not touching the bottom of the pool (O’Hara and Co., Ltd., Tokyo, Japan). Video recordings for 5 min were measured with the EthoVision XT video imaging system (n = 8) [25].

### 2.8. Sucrose Preference Test

Anhedonic behavior was measured using the sucrose consumption test. After a 24-h water deprivation period, each animal was exposed to two bottles, one containing tap water (200 mL) and the other containing sucrose solution (1%) (200 mL). The amounts of sucrose solution and water consumed after 24 h were recorded [26]. The positions of the two bottles were changed randomly.

Sugar preference value = sugar consumption/(sugar consumption + water consumption) × 100%

### 2.9. RNA Isolation and cDNA Synthesis

Homogenized tissue samples were isolated using the High Pure RNA Isolation kit (Cat. No: 11828665001) (Roche, Basel, Switzerland). RNA samples for cDNA were performed using the Transcriptor First Strand cDNA Synthesis kit (Cat. No: 04896866001) (Roche, Basel, Switzerland) according to the specified protocol. 

### 2.10. Real-Time PCR

Real-time PCR was performed using primers constructed for rat Slc1a3 Rn01402419_m1, rat Slc1a2 Rn01275814_m1, rat Arl6ip5 Rn01420961_m1, rat Glul Rn01483107_m1 and rat β-actin Rn00667869_m1. Values were calculated by calculating 2−ΔΔCt of Real Time PCR CT values [27].

### 2.11. In Vivo Voltammetry

Rats weighing 250–300 g at the end of 60 days were selected for in vivo voltammetry (n = 3). The Fast Analytical Detection Technology (FAST 16) system was used to simultaneously monitor the rapid changes of glut in the synaptic gap. First of all, the calibration of the silver reference electrode and the S2 electrode with serial number A537 was provided. Bregma of the cornu ammonis 3 (CA3) and dorsal subiculum (DS) of the hippocampus, which are brain regions associated with depression, are accepted as 0 in the x and y axes, and the coordinates are DS (−3.1 on the x-axis/−6 on the y-axis/3 on the z-axis). CA3 (+3.2 in the x-axis/−3.6 in the y-axis/3.5 in the z-axis) was determined [28]. Finally, the T_80_ value was used for statistical analysis.

### 2.12. Glutathione (GSH), Lactate Dehydrogenase (LDH) and Acetylcholinesterase (AChE) Analysis

The determination of glutathione, lactate dehydrogenase, and acetylcholinesterase levels in brain tissue samples by the ELISA method rat glutathione (GSH) (Cat. No: E-EL-R0026), lactate dehydrogenase (LDH) (Cat. No: E-EL-R2547) and acetylcholinesterase AChE (Cat. No: SL0027Ra) ELISA were performed using the kit. The determinations were made in the ELISA device at a wavelength of 450 nm as a reference.

### 2.13. Histopathological Examinations

Tissue samples were fixed in 10% formalin solution for 48 h. The preparations prepared for histopathological examination were stained with hematoxylin-eosin (H&E) and examined by light microscopy (Leica DM 1000, Leica Microsystems, Wetzlar, Germany). The percent of damaged neuron cells in the shaded area is given as a percentage.

### 2.14. Immunohistochemical Studies

All sections taken on the adhesive (poly-L-Lysin) slides for immunoperoxidase examination were passed through the xylol and alcohol series. 8-hydroxy-2’-deoxyguanosine (8 OHdG), glial fibrillar acidic protein (GFAP), neuronal nitric oxide synthase (nNOS), (Cat. No: ab68428, dilution 1/100; UK). Followed according to the immunohistochemistry kit procedure (Abcam HRP/DAB Detection IHC kit). 3-3’ Diaminobenzidine (DAB) was used as chromogen. Floor painting was done with hematoxylin. Preparations prepared for histopathological examination were examined with a laser scanning confocal microscope (Zeiss LSM 710, Carl Zeiss AG, Oberkochen, Germany). 8-OHdG and GFAP expression is defined by increased brown colors in tissue; nNOS is defined by the increase in radiolucency ratio. For quantitative analysis of IHC and immunofluorescence staining, the mean immunoreactivity intensity was determined using ImageJ software (ImageJ). After color deconvolution, the images were inverted. Average pixel density was measured with ImageJ. Five areas from each group were evaluated.

### 2.15. Statistical Analysis

Tukey’s least significant difference (LSD) test was used following one-way analysis of variance (one-way ANOVA) to evaluate the findings obtained in behavioral changes that occur with stress. Statistical analyzes were also used by using ‘SPSS 22.0’ (IBM, Armonk, NY, USA) and result in less than or equal to *p* < 0.05 and *p* < 0.001 were considered statistically significant. The Kruskal Wallis method was used to evaluate the density of immunoreactivity between nonparametric groups. *p* < 0.05 was considered statistically significant. 

## 3. Results

### 3.1. Validation of Mild Stress Model of Rats

**Figure 2:**
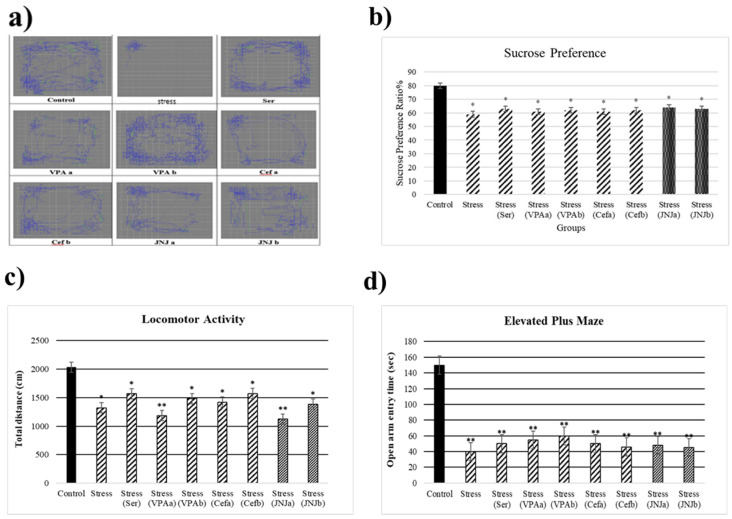
Behavioral performances of experimental groups exposed to stress 30 days. Locomotor activity: (**a**) schematic of traveled area, (**b**) Sucrose preference ratio, (**c**) locomotor activity, (**d**) Elevated plus-maze test: open arm entry time (s). One-way ANOVA, * *p*  <  0.05, ** *p*  <  0.001 compared with the Control group.

At the end of the 30th day, the stress model was evaluated (Figure 2). The average sucrose consumption ratio in the control group was 80%, while in the Stress groups it significantly decreased to 59, 63, 61, 62, 61, 62, 64 and 63% respectively (*p* < 0.05) (Figure 2b). After 30 days of exposure to stress the locomotor activity in the model groups significantly decreased (1323, 1569, 1185, 1483, 1423, 1574, 1125 and 1386 cm, respectively) compared to the control group (2034 cm) (*p* < 0.05) (Figure 2c). The average time spent in the open arm in the control group was 150 sec, while a significant decrease in this parameter in the Stress groups was observed (40, 50, 55, 60, 50, 46, 48 and 45 sec) (*p* < 0.001) (Figure 2d). 

### 3.2. JNJ Treatment Increases Locomotor Activity in a Mild Stress Model of Rats

It was observed that the locomotor activity was significantly decreased in the Stress group (1279 cm) compared with the control group (2493 cm) (Figure 2a,b) (*p* < 0.001). The treatment with Ser, VPA and Cef significantly increased the locomotor activity of mild stress rats compared with the Stress group, the effect being dose-dependent for the last two drugs. The treatment with JNJa (1809 cm) and JNJb (2111 cm) increased the locomotor activity of mild stress rats, the effect being similar to those produced by the known antidepressant drugs. In the case of JNJ the effect was dose-dependent, the locomotor activity in group JNJb being significantly increased compared with the one obtained for the group JNJa. 

### 3.3. JNJ Treatment Increases Locomotor Activity in a Mild Stress Model of Rats

**Figure 3:**
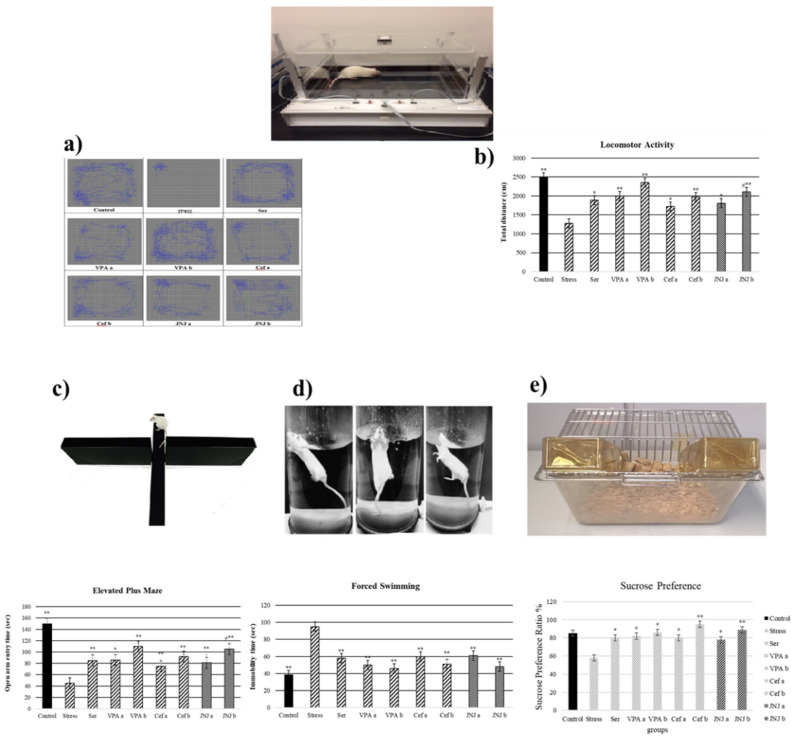
Behavioral performances of experimental groups exposed to stress 30 days after treatment. Locomotor activity: (**a**) schematic of travel area. (**b**) distance traveled. (**c**) Elevated plus-maze test: open arm entry time (s). (**d**) Forced swimming: immobility time (s). (**e**) Sucrose preference amount consumption (%). One-way ANOVA, * *p*  <  0.05, ** *p*  <  0.001 compared with the Stress group.; ≠ *p*  <  0.05 compared with JNJa.

It was observed that the locomotor activity was significantly decreased in the Stress group (1279 cm) compared with the control group (2493 cm) (Figure 3a,b) (*p* < 0.001). The treatment with Ser, VPA and Cef significantly increased the locomotor activity of mild stress rats compared with the Stress group, the effect of the last two drugs being dose-dependent. The treatment with JNJa (1809 cm) and JNJb (2111 cm) increased the locomotor activity of mild stress rats, the effect being similar to those produced by the known antidepressant drugs. In the case of JNJ the effect was dose-dependent, the locomotor activity in group JNJb being significantly increased compared with the one obtained for the group JNJa. 

### 3.4. JNJ Treatment Increases Time Spent in Open Arm in Elevated plus Maze Test in a Mild Stress Model of Rats

The time spent by the rats in the open arm in a 5 min period is presented in Figure 3c in seconds (s). The average time spent in the open arm in the control group was 150 s, while a significant decrease in this parameter in the Stress group was observed (45 s). The administration of antidepressant drugs significantly increased the time spent in the open arm compared with the Stress group to 85 s for Ser group, 86 s and 110 s for VPAa and VPAb groups (*p* < 0.001) and 75 s (*p* < 0.05) and 92 s (*p* < 0.001) for Cefa and Cefb groups. 

The treatment with JNJa (81 s) and JNJb (105 s) significantly increased the time spent in the open arm compared with the Stress group (*p* < 0.001), the effect being similar to those produced by the known antidepressant drugs. In the case of JNJ, the effect was dose-dependent, the time spend in the open arm in group JNJb being significantly increased compared with the one obtained for the group JNJa, showing that the H4 receptor antagonist JNJ significantly decreases anxiety and stress levels.

### 3.5. JNJ Treatment Decreases Immobility Time in Forced Swimming Test in a Mild Stress Model of Rats

In the 5-min forced swimming test, the immobility times in seconds were presented in Figure 3d. The average time of immobility in the control group was 38 s, while the Stress group showed a significant increase to 95 s (*p* < 0.001). The treatment with Ser, VPA a-b, and Cef a-b significantly decreased the immobility time compared with the Stress group to 58, 50–46, and 60–51 s (*p* < 0.001), respectively. The treatment with H4 receptor antagonist JNJ significantly decreased the immobility time compared with the Stress group to 61 s in JNJa and 48 s in JNJb (*p* < 0.001), without a significant difference between the doses administered. 

### 3.6. JNJ Treatment Increase Sucrose Preference in a Mild Stress Model of Rats

The sucrose preference test, with the consumption of sucrose in water is shown in Figure 3e. The average sucrose consumption ratio in the control group was 85%, while the Stress group showed a significant decrease to 58% (*p* < 0.05). The treatment with Ser, VPA a-b, and Cef a-b significantly increased the sucrose consumption ratio compared with the Stress group to 80, 82–86, 80–95 (*p* < 0.05), respectively.

The treatment with H4 receptor antagonist JNJ significantly increased the sucrose preference ratio compared with the Stress group to 78% in JNJa (*p* < 0.05) and 89% in JNJb (*p* < 0.001), without a significant difference between the doses administered. 

### 3.7. JNJ Treatment Decreased T80 Time of Glut Reuptake in CA3 and DS Regions in a Mild Stress Model of Rats

**Figure 4:**
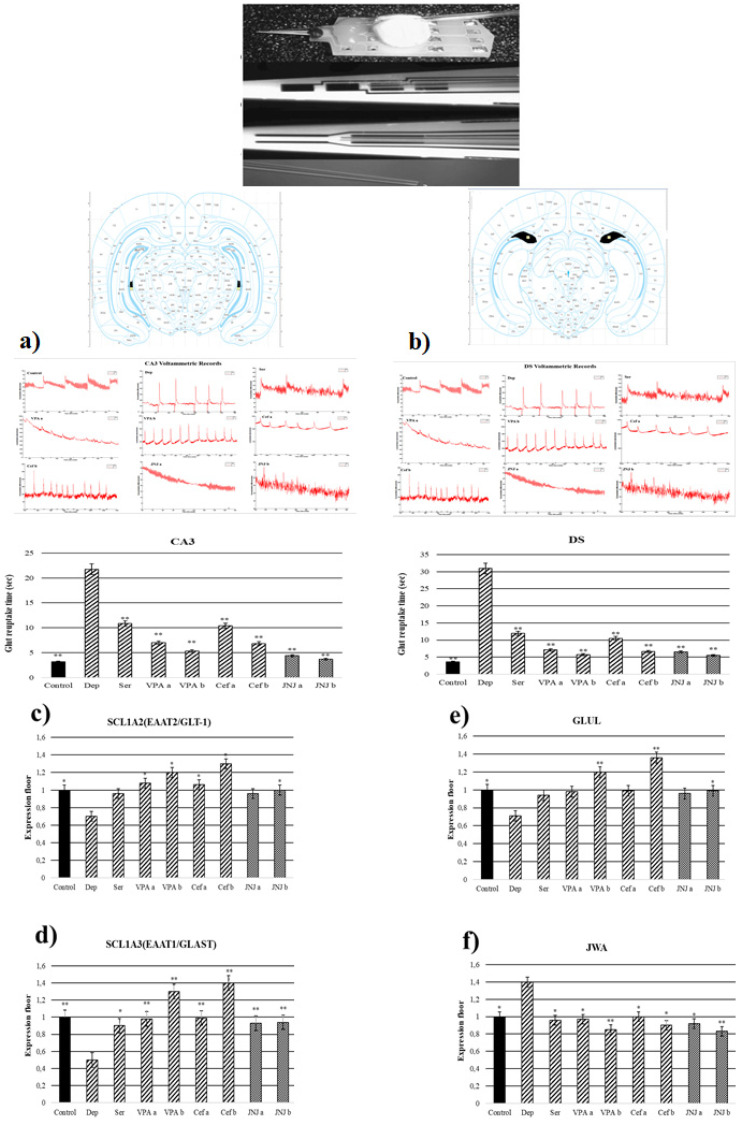
Glut reuptake times and gene expressions of experimental groups 30 days after treatment. (**a**) CA3 voltammetric records and glut reuptake time (s). (**b**) DS voltammetric records and glut reuptake time (s) (**c**) SLC1A2 gene expression. (**d**) GLUL gene expression (**e**) SLC1A3 gene expression. (**f**) JWA gene expression. One-way ANOVA, * *p*  <  0.05, ** *p*  <  0.001 compared with the Stress group.

T80 time of the glut reuptake was measured in CA3 and DS regions (Figure 4a,b). In the Stress group, the T80 times of the glut reuptake in the CA3 region was significantly increased to 21.76 s compared with the control group (3.17 s). The treatment with known antidepressant drugs significantly decreased the T80 time in CA3 compared with the Stress group to 10.83, 7–5, and 10.37–6.85 s in Ser, VPA a-b, and Cef a-b, respectively. The treatment with JNJ significantly decreased the T80 time in a dose dependent-manner till 4.37 s in the JNJa group and 3.67 s in the JNJb group (*p* < 0.001). The same effects are observed also in the DS region. In the stress group, the T80 time was significantly increased to 30.94 s compared with the control group, where the T80 time was 3.59 s. The treatment with the Ser, VPA a-b, and Cef a-b significantly decreased the T80 time of glut reuptake compared with the Stress group to 12, 7.12–5.70 and 10.5–6.65 s, respectively. The same results were also observed in the groups treated with JNJ, where the effect was dose-dependent 6.55 s for JNJa and 5.49 s for JNJb, but without reaching the statistical difference between the two concentrations used. 

### 3.8. JNJ Treatment Increased SLC1A2, SLC1A3, and GLUL Gene Expression and Decreased JWA Gene Expression in a Mild Stress Model of Rats

In the Stress group, the SLC1A2 gene expression in the hippocampus region was significantly downregulated compared with the control group (*p* < 0.05). In the positive control groups, only VPA and Cef determined a significant up-regulation of SLC1A2 gene expression compared with the Stress group in a dose-dependent manner (*p* < 0.05). The treatment with JNJ significantly up-regulates the SLC1A2 gene expression only in the higher dose regiment (group JNJb). A significant difference in the SLC1A2 gene expression between the JNJa group and the JNJb group was not seen (*p* > 0.05) (Figure 4c).

In the Stress group, we observed a down-regulation of SLC1A3 gene expression compared with the control group (*p* < 0.05). The treatment with Ser, VPA, and Cef significantly up-regulated the SLC1A3 gene expression in a dose-dependent manner almost to the level of the control group. Also, the treatment with JNJ significantly up-regulates the SLC1A3 gene expression almost to the level of the control group (*p* < 0.001), but the effect was not dose-dependent (Figure 4d). 

In the case of GLUL gene expression, in the Stress group we observed a significant down-regulation compared with the control group. Only the treatment with a higher dose of VPA (group VPAb) and Cef (group Cefb) succeeded in significantly up-regulating the level of the GLUL gene expression compared with the Stress group levels. The treatment with the highest concentration of JNJ (group JNJb) also succeeded in significantly up-regulating the GLUL gene expression compared with the Stress group level, reaching the level of the Control group (Figure 4e). 

In the Stress group, we observed a significant up-regulation of JWA gene expression compared with the control group. The treatment with known antidepressant drugs such as Ser, VPA, and Cef significantly down-regulated the JWA gene expression compared with the Stress group levels. The same was observed also after the treatment with JNJ, the effect being dose-dependent, but without a significant difference between the JWA expression level in the JNJa group and JNJb group (*p* > 0.05) (Figure 4f).

### 3.9. JNJ Treatment Increase GSH Levels in Brain Tissue in a Mild Stress Model of Rats

**Figure 5:**
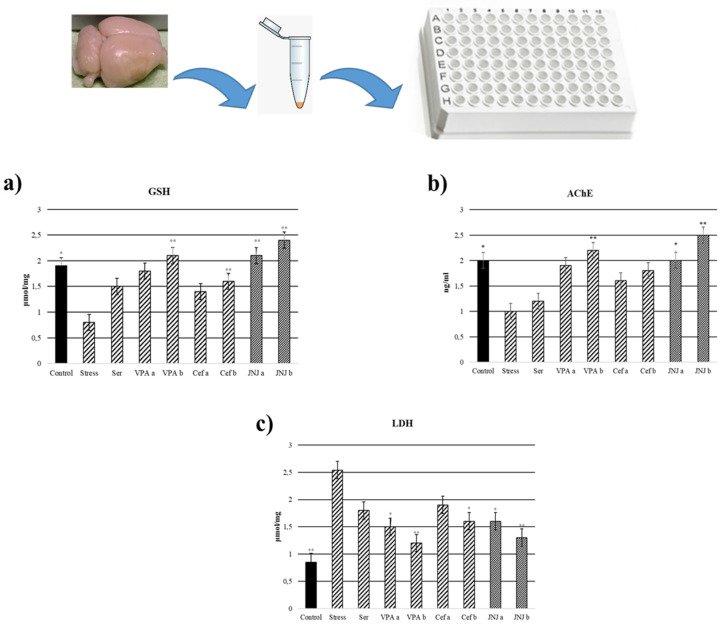
GSH, LDH, and acetylcholinesterase levels 30 days after treatment. (**a**) GSH levels (µmol/mg). (**b**) Acetylcholinesterase levels (ng/mL). (**c**) LDH levels (µmol/mg) after treatment. One-way ANOVA, * *p*  <  0.05, ** *p*  <  0.001 compared with the Stress group.

In the Stress group, the levels of GSH in brain tissues significantly decreased to 0.8 µmol/mg compared with the control group (1.9 µmol/mg) (*p* < 0.05). In the case of positive control groups, only the highest concentration of VPA (VPAb group, 2.1 µmol/mg) and Cef (Cefb group, 1.6 µmol/mg) significantly increased the GSH level compared with the Stress group. The treatment with JNJ significantly increased the GSH levels compared with the Stress group to 2.1 µmol/mg in the JNJa group and 2.4 µmol/mg in the JNJb group (*p* < 0.001). There is no significant difference between JNJ groups (*p* > 0.05) (Figure 5a).

### 3.10. JNJ Treatment Decreased LDH Levels in Brain Tissue in a Mild Stress Model of Rats

LDH levels in brain tissues were significantly increased in the Stress group (2.54 µmol/mg) compared with the control group (0.85 µmol/mg) (*p* < 0.001). In the positive control group, only VPA in both doses (1.5 µmol/mg for VPAa and 1.2 µmol/mg for VPAb) and the highest dose of Cef (1.6 µmol/mg for Cefb) significantly decreased the LDH levels compared with the Stress group. The treatment with both concentrations of JNJ significantly decreased the LDH levels to 1.6 µmol/mg for JNJa (*p* < 0.05) and 1.3 µmol/mg for JNJb (*p* < 0.001) compared with the Stress group levels. There was no significant difference between JNJ groups (*p* > 0.05) (Figure 5b).

### 3.11. JNJ Treatment Increased AChE Activity in Brain Tissue in a Mild Stress Model of Rats

AChE activity in brain tissues significantly decreased in the Stress group (1 ng/m) compared with the control group (2 ng/m) (*p* < 0.001). In the positive control groups, a slight increase in AChE activity compared with the Stress group was observed, but the statistical significance was obtained only for the higher concentration of VPA (the value for the VPAb group was 2.2 ng/mL). The treatment with JNJ significantly increased AChE activity compared with the Stress group to 2 ng/mL for JNJa (*p* < 0.05) and 2.5 ng for JNJb (*p* < 0.001). There was no significant difference between JNJ groups (*p* > 0.05) (Figure 5c).

### 3.12. JNJ Treatment Prevent Severe Histopathological Degeneration of Brain Tissue Produced by Mild Stress

**Figure 6:**
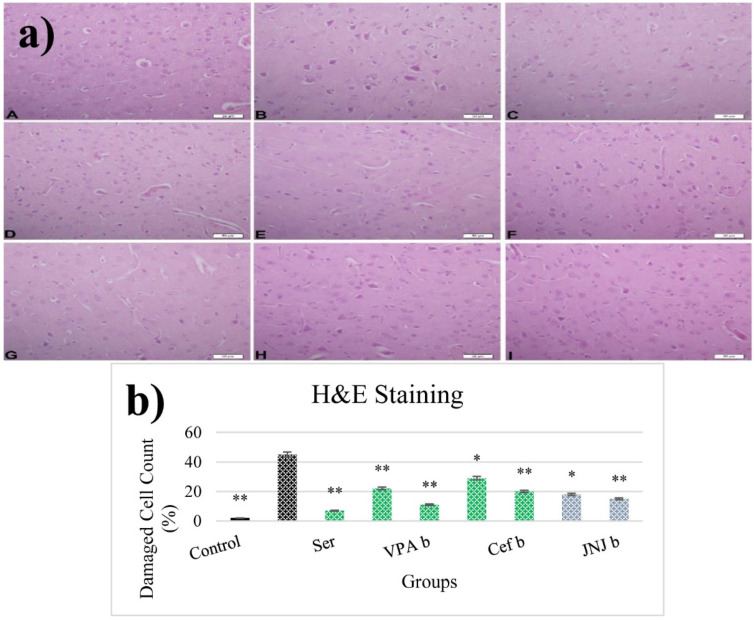
(**a**) H&E images of experimental groups. Histopathological images of A (Control), B (Stress), C (Ser), D (VPAa), E (VPAb), F (Cefa), G(Cefb), H (JNJa), and I (JNJb). (**b**) The number of damaged cells is given as a percentage after quantitative determination. * *p*  <  0.05, ** *p*  <  0.001 compared with the Stress group.

**Figure 7:**
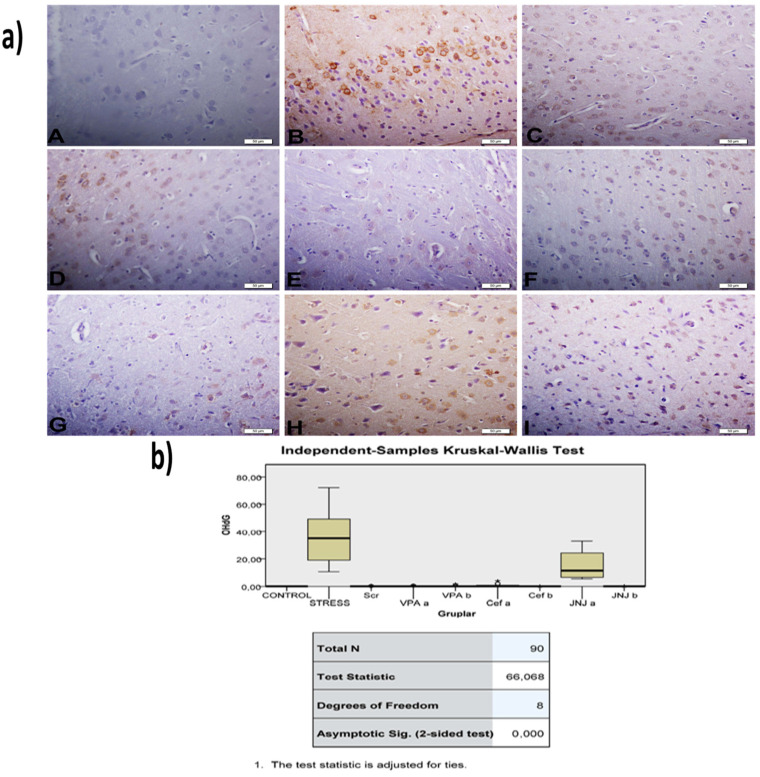
(**a**) 8-OHdG images of experimental groups. Histopathological images of A (Control), B (Stress), C (Ser), D (VPAa), E (VPAb), F (Cefa), G(Cefb), H (JNJa), and I (JNJb). IHC-P—immunohistochemistry-paraffin protocol. (**b**) Quantitative analysis of IHC, immunofluorescence staining, and mean immunoreactivity intensity was determined using ImageJ software (ImageJ). *p* < 0.05 was considered statistically significant.

**Figure 8:**
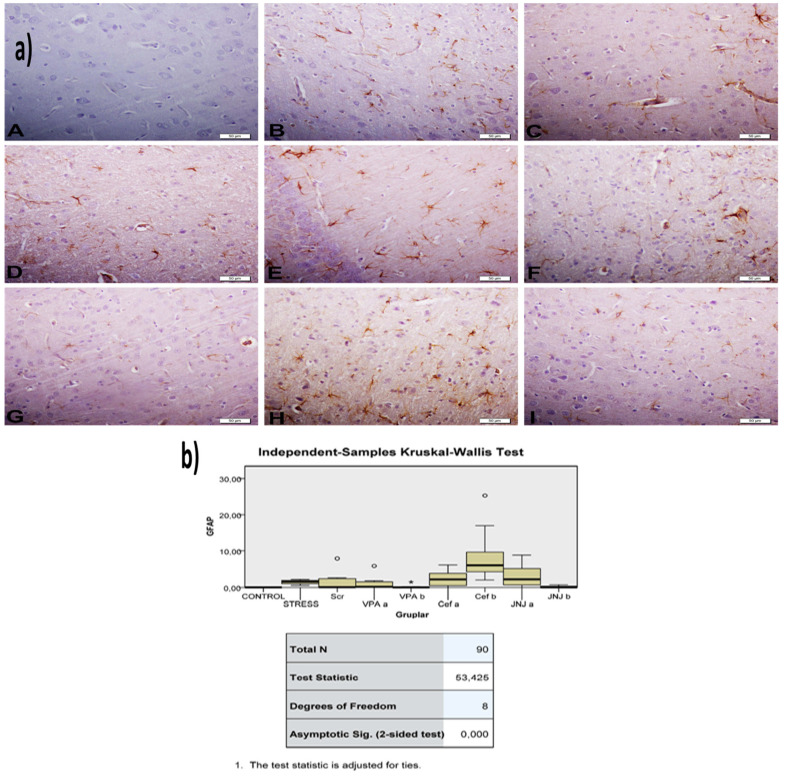
(**a**) GFAP images of experimental groups. Histopathological images of A (Control), B (Stress), C (Ser), D (VPAa), E (VPAb), F (Cefa), G(Cefb), H (JNJa), and I (JNJb). IHC-P—immunohistochemistry-paraffin protocol. (**b**) Quantitative analysis of IHC and immunofluorescence staining, mean immunoreactivity intensity was determined using ImageJ software (ImageJ). *p* < 0.05 was considered statistically significant.

**Figure 9:**
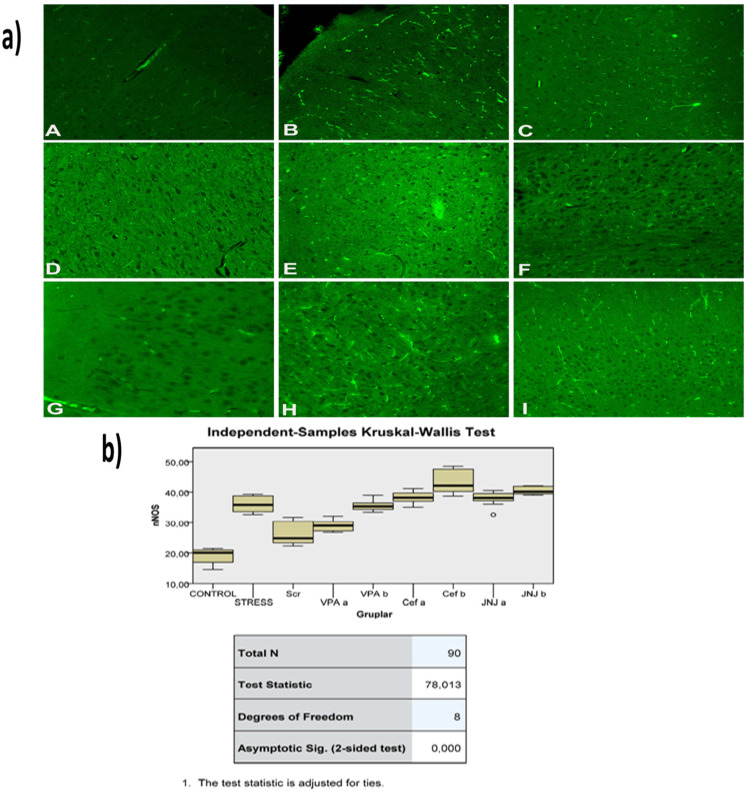
(**a**) nNOS images of experimental groups. Histopathological images of A (Control), B (Stress), C (Ser), D (VPAa), E (VPAb), F (Cefa), G(Cefb), H (JNJa), and I (JNJb). IHC-P—immunohistochemistry-paraffin protocol; IF—immunofluorescent staining. (**b**) Quantitative analysis of IHC, immunofluorescence staining, and mean immunoreactivity intensity was determined using ImageJ software (ImageJ). *p* < 0.05 was considered statistically significant.

The histopathological evaluation of brain tissue through H&E staining revealed that in the control group the structure was normal. The exposure to stress caused very severe degeneration in neuron cells, necrosis, and hyperemia in the parenchymal and meningeal vessels in the Stress group. The treatment with Ser, VPA and Cef significantly decreased the degeneration of neuron cells, necrosis and hyperemia. The effect was dose-dependent for VPA and Cef, with the highest dose of Cef being similar to Ser. The treatment with the H4 receptor antagonist JNJ also decreased the histopathological changes produced by the exposure to stressful stimuli (Figure 6). The evaluation of immunohistochemical expression of 8-OHdG (Figure 7) and GFAP (Figure 8), and immunofluorescence of nNOS (Figure 9) in the brain tissue revealed that in the Stress group an up-regulation of these markers and the administration of known antidepressant drugs decreased 8-OHdG (Figure 7), nNOS (Figure 9) and, GFAP (Figure 8) immune-expression in the Stress group, the effects the being dose-dependent. In terms of GFAP expression, there was a decrease in the VPA b and JNJ b groups compared to the stress group, but this decrease was not statistically significant (Figure 8). Compared to the stress group, 8-OHdG immunoreactivity showed a statistically significant decrease in the Scr, JNJ b, Cef a, Cef b, VPA a and VPA b groups, except for the JNJ a group (*p* < 0.05) (Figure 7). nNOS expression was evaluated by the immunofluorescence staining method. Although there was a decrease in the immunoreactivity density in the other groups compared to the stress group, no statistically significant difference was observed (Figure 9).

## 4. Discussion

The effects of conventional antidepressant drugs like Ser and VPA are discussable (only 50% of patients show a progression) [5,6,29]. In addition, those drugs require weeks to months of use prior to the alleviation of symptoms [30]. The neurotransmitter receptor has gained importance in the monoamine hypothesis in depression progress [31,32]. Studies conducted in line with this hypothesis have shown that glut uptake is reduced in the hippocampus, striatum, and prefrontal cortex. In particular, they reported that hopeless animals expressed significantly lower levels of GLT1 in the hippocampus and cortex compared to offspring that did not display helpless behavior [8,10]. glut release and reuptake play a key role in the pathophysiology of depression [33,34,35,36]. 

It is also known that glutamatergic nerves in the hippocampus region are modulated by histaminergic afferents [14,37] We found a modulation role of the H4 antagonist with glut receptor-transporters in chronic depression. In our experiment, it has been shown that blocking the H4 receptor leads to increased neuron cell viability and improvement in behavioral ability due to glut.

Previously, it was observed that the locomotor activity of the depressed group decreased approximately 40% compared to the control group [29]. In our study, locomotor activity shows a 50% decrease in the depression group depending on neurodegeneration. Ser and VPA agents used for positive control show an increase in locomotor activity while the Cef activity finding is debatable. JNJ increased locomotor activity similarly to the control group. In addition, JNJ improved movement ability better than Ser and VPA (low dose). Pan et al., in their study, measured the locomotor abnormalities that developed in animals after inducing vestibular lesions using orexin-A. They showed that JNJ (20 mg/kg, i.p.), which they used for the treatment of vestibular damage, significantly reduced abnormal locomotor activity [38]. Elevated plus-maze results also correlate with locomotor activity. The increasing dose of drugs has been shown to prolong open-arm stays. Many studies have shown that the duration of staying in the closed arm is longer than the open arm in chronic depression due to anxiety and stress [8,9,29]. JNJ, by affecting the glut transporter (EAAT1 and EAAT2 gene expression level increased 50%) in the hippocampus, CA3 and DS regions decreased glut reuptake time and decreased glut neurotoxicity. Real-time neurotransmitter monitoring by voltammetry technique showed Stress group glut reuptake time increased to 21 sn while in the JNJ group time decreased to 4.3 and 3.6 sn respectively. All this data shows that the H4 antagonist decreased glut-dependent neurotoxicity by affecting the transporter expression level and increasing the reuptake activity. In addition, the DS region compared to CA3 is sensitive to depression-dependent glut toxicity. The protective effect of JNJ_b_ on DS is more obvious because of the cell population ratio. On the other hand, the Stress group immobility duration in the forced swimming experiment shows an increase. However, it was observed that VPA, Ser, and JNJ delayed transient to this passive (energy conserving) behavior style. In addition, JNJ_b_ improved swimming ability better than Ser, VPA_a_, Cef_a_, and Cef_b_. It has been reported that the consumption of sucrose water increases as a result of the increase in the sense of pleasure in healthy animals [39]. In our study, it was found that the treatment groups increased their consumption of sucrose water compared to the depression group. However, the effect of VPA and JNJ on anhedonic behavior was examined for the first time in our study.

Glut transporters are accepted as a cause of depressive disorders; EAAT1 (43%) [40] and EAAT2 (50%) [41] expression levels were found to be significantly lower than the control group. However, some studies reported increased glut release and receptor expression associated with chronic stress in the hippocampus [42]. The JWA gene (depression increased near to 40% gene expression) overexpression has been found to inhibit neurite outgrowth of neuronal cells [43]. Wang et al. showed that JWA deletion in astrocytes impairs glut uptake by reducing GLT-1 expression. They also reported that JWA induced GLT-1 expression through the ERK/Akt and CREB cascade and reactivated astrocytes with STAT3 [44]. Zink et al. reported that in the learned helplessness stress paradigm study, the GLT-1 expression level decreased by 50% compared to the healthy group, while this rate did not change in GLAST [10]. These data demonstrated that JWA plays a neuroprotective role in astrocytes. A high glutamine/glut ratio was also observed in this study, which could be explained by the disruption of the glut/glutamine cycle. 

The oxidative damage in the brain tissues of rats depends on depression-glut toxicity, causing a significant decrease in GSH levels [45,46]. It was proved that glut toxicity decreased GSH levels by the induction of oxidative stress [47,48,49]. Also, Zafir et al. showed that LDH levels in the brain tissues of rats increased with cellular damage under restraint stress [50]. According to the Cai et al. study, low GSH levels were reported in the post-stroke depression model compared to the sham group depending on neurodegeneration [51], which was confirmed in our study.

Our observation shows a significant decrease in GSH levels in the brain tissues of depressed rats, while an increase was observed in the treatment groups due to increasing doses. The JNJ_b_ shows promise in preventing free oxygen radicals and increasing GSH levels. The damaged cell plasma membranes must be a result of glut leakage. Therefore, JNJ can be used to prevent neurotoxicity, inhibit membrane phospholipase activation and free radical formation, and minimize membrane disruption.

The abnormal surge of AChE directly affects acetylcholine metabolism and thus impairs neurotransmission in the brain [52]. Previous studies have found that increased acetylcholine levels in the hippocampus are positively correlated with increased depression [53]. Another study showed that AChE expression increased by 52% and exhibited depression-like behavior in animals [54]. Excess AChE emerges under conditions of stress. Subsequently, the chronic blockade of the AChE with specific inhibitors selectively impaired glutamatergic functions and excitatory synaptic structures independently [55]. In contrast, other studies have shown that AChE inhibitors increase the depressive phenotype [56]. In our study, we observed a 50% decrease in AChE activity in the brain tissues of depressed rats, while we observed a 25% increase in AChE activities in the treatment groups due to increasing doses. 

Neuronal injury is excitotoxicity, mediated by massive glut release, which leads to the excessive stimulation of N-methyl-D aspartate receptors and an influx of calcium [57,58,59]. Calcium overload leads to the activation of nNOS. Thus, nNOS catalyzes the oxidation of L-arginine to produce NO, which leads to excitotoxicity and neuronal cell death [60]. Also, an increase was observed in nNOS levels in the hippocampus due to glut toxicity induction and oxidative stress [61,62]. It was shown that the 8-OHdG and GFAP expression level was increased due to an increase in inflammation and oxidative stress level in major depressive disorder [63,64,65,66]. Immunopathological evaluation of nNOS, GFAP and 8-OHdG proteins expression in treatment groups showed a slight to moderate decrease in Ser, VPA_a_ and JNJ_a_ in the Cef group, to significant decrease in VPA_b_ and JNJ_b_ treatment. 

## 5. Conclusions

In conclusion, it has been shown that blocking the H4 receptor leads to increased neuron cell viability and improvement in behavioral ability due to glut excitotoxicity. Therefore, JNJ can be used to prevent neurotoxicity, inhibit membrane phospholipase activation and free radical formation, and minimize membrane disruption. In line with our findings, results have been obtained that show that JNJ will contribute to the effective prevention and treatment of depression.

## Data Availability

The data presented in this study are available on request from the corresponding authors. The data are not publicly available due to privacy and ethical restrictions.

## References

[1] GBD 2019 Diseases and Injuries Collaborators (2020). Global burden of 369 diseases and injuries in 204 countries and territories, 1990–2019: A systematic analysis for the Global Burden of Disease Study 2019. Lancet.

[2] Santomauro D.F., Herrera A.M.M., Shadid J., Zheng P., Ashbaugh C., Pigott D.M., Abbafati C., Adolph C., Amlag J.O., Aravkin A.Y. (2021). Global prevalence and burden of depressive and anxiety disorders in 204 countries and territories in 2020 due to the COVID-19 pandemic. Lancet.

[3] Jesulola E., Micalos P., Baguley I.J. (2018). Understanding the pathophysiology of depression: From monoamines to the neurogenesis hypothesis model-Are we there yet?. Behav. Brain Res..

[4] Dodd S., Bauer M., Carvalho A.F., Eyre H., Fava M., Kasper S., Kennedy S.H., Khoo J.P., Lopez Jaramillo C., Malhi G.S. (2021). A clinical approach to treatment resistance in depressed patients: What to do when the usual treatments don’t work well enough?. World J. Biol. Psychiatry.

[5] Krishnan V., Nestler E.J. (2008). The molecular neurobiology of depression. Nature.

[6] Nestler E.J., Barrot M., DiLeone R.J., Eisch A.J., Gold S.J., Monteggia L.M. (2002). Neurobiology of depression. Neuron.

[7] Schroeder M., Hillemacher T., Bleich S., Frieling H. (2012). The Epigenetic Code in Depression: Implications for Treatment. Clin. Pharmacol. Ther..

[8] Almeida R.F., Thomazi A.P., Godinho G.F., Saute J.A.M., Wofchuk S.T., Souza D.O., Ganzella M. (2010). Effects of Depressive-Like Behavior of Rats on Brain Glutamate Uptake. Neurochem. Res..

[9] De Vasconcellos-Bittencourt A.P., Vendite D.A., Nassif M., Crema L.M., Frozza R., Thomazi A.P., Nieto F.B., Wofchuk S., Salbego C., da Rocha E.R. (2011). Chronic stress and lithium treatments alter hippocampal glutamate uptake and release in the rat and potentiate necrotic cellular death after oxygen and glucose deprivation. Neurochem. Res.

[10] Zink M., Vollmayr B., Gebicke-Haerter P.J., Henn F.A. (2010). Reduced expression of glutamate transporters vGluT1, EAAT2 and EAAT4 in learned helpless rats, an animal model of depression. Neuropharmacology.

[11] Altshuler L.L., Abulseoud O.A., Foland-Ross L., Bartzokis G., Chang S., Mintz J., Hellemann G., Vinters H.V. (2010). Amygdala astrocyte reduction in subjects with major depressive disorder but not bipolar disorder. Bipolar Disord..

[12] Bernard R., Kerman I.A., Thompson R.C., Jones E.G., Bunney W.E., Barchas J.D., Schatzberg A.F., Myers R.M., Akil H., Watson S.J. (2011). Altered expression of glutamate signaling, growth factor, and glia genes in the locus coeruleus of patients with major depression. Mol. Psychiatr..

[13] Miguel-Hidalgo J.J., Waltzer R., Whittom A.A., Austin M.C., Rajkowska G., Stockmeier C.A. (2010). Glial and glutamatergic markers in depression, alcoholism, and their comorbidity. J. Affect. Disord..

[14] Rodriguez F.J., Lluch M., Dot J., Blanco I., RodriguezAlvarez J. (1997). Histamine modulation of glutamate release from hippocampal synaptosomes. Eur. J. Pharmacol..

[15] Lu C.W., Lin T.Y., Chang C.Y., Huang S.K., Wang S.J. (2017). Ciproxifan, a histamine H-3 receptor antagonist and inverse agonist, presynaptically inhibits glutamate release in rat hippocampus. Toxicol. Appl. Pharm..

[16] Karpati A., Yoshikawa T., Nakamura T., Iida T., Matsuzawa T., Kitano H., Harada R., Yanai K. (2018). Histamine elicits glutamate release from cultured astrocytes. J. Pharmacol. Sci..

[17] Leurs R., Chazot P.L., Shenton F.C., Lim H.D., de Esch I.J. (2009). Molecular and biochemical pharmacology of the histamine H4 receptor. Br. J. Pharmacol..

[18] Terzioglu Bebitoglu B., Oguz E., Gokce A. (2020). Effect of valproic acid on oxidative stress parameters of glutamate-induced excitotoxicity in SH-SY5Y cells. Exp. Ther. Med..

[19] Kobayashi H., Iwata M., Mitani H., Yamada T., Nakagome K., Kaneko K. (2012). Valproic acid improves the tolerance for the stress in learned helplessness rats. Neurosci. Res..

[20] Tikhonova M.A., Amstislavskaya T.G., Ho Y.J., Akopyan A.A., Tenditnik M.V., Ovsyukova M.V., Bashirzade A.A., Dubrovina N.I., Aftanas L.I. (2021). Neuroprotective Effects of Ceftriaxone Involve the Reduction of Abeta Burden and Neuroinflammatory Response in a Mouse Model of Alzheimer’s Disease. Front. Neurosci..

[21] Lee S.G., Su Z.Z., Emdad L., Gupta P., Sarkar D., Borjabad A., Volsky D.J., Fisher P.B. (2008). Mechanism of ceftriaxone induction of excitatory amino acid transporter-2 expression and glutamate uptake in primary human astrocytes. J. Biol. Chem..

[22] Zhou M., Liu Z., Yu J., Li S.M., Tang M., Zeng L., Wang H.Y., Xie H., Peng L., Huang H.J. (2018). Quantitative Proteomic Analysis Reveals Synaptic Dysfunction in the Amygdala of Rats Susceptible to Chronic Mild Stress. Neuroscience.

[23] Tsatsakis A., Tyshko N.V., Docea A.O., Shestakova S.I., Sidorova Y.S., Petrov N.A., Zlatian O., Mach M., Hartung T., Tutelyan V.A. (2019). The effect of chronic vitamin deficiency and long term very low dose exposure to 6 pesticides mixture on neurological outcomes-A real-life risk simulation approach. Toxicol. Lett..

[24] Tsoukalas D., Zlatian O., Mitroi M., Renieri E., Tsatsakis A., Izotov B.N., Burada F., Sosoi S., Burada E., Buga A.M. (2021). A Novel Nutraceutical Formulation Can Improve Motor Activity and Decrease the Stress Level in a Murine Model of Middle-Age Animals. J. Clin. Med..

[25] Han X., Shao W., Liu Z., Fan S., Yu J., Chen J., Qiao R., Zhou J., Xie P. (2015). iTRAQ-based quantitative analysis of hippocampal postsynaptic density-associated proteins in a rat chronic mild stress model of depression. Neuroscience.

[26] Uchida S., Hara K., Kobayashi A., Otsuki K., Yamagata H., Hobara T., Suzuki T., Miyata N., Watanabe Y. (2011). Epigenetic Status of Gdnf in the Ventral Striatum Determines Susceptibility and Adaptation to Daily Stressful Events. Neuron.

[27] Livak K.J., Schmittgen T.D. (2001). Analysis of relative gene expression data using real-time quantitative PCR and the 2(T)(-Delta Delta C) method. Methods.

[28] Paxinos G., Watson C. (2006). The Rat Brain in Stereotaxic Coordinates.

[29] Hu C.L., Luo Y., Wang H., Kuang S.N., Liang G.J., Yang Y., Mai S.S., Yang J.Q. (2017). Re-evaluation of the interrelationships among the behavioral tests in rats exposed to chronic unpredictable mild stress. PLoS ONE.

[30] Trivedi M.H., Rush A.J., Wisniewski S.R., Nierenberg A.A., Warden D., Ritz L., Norquist G., Howland R.H., Lebowitz B., McGrath P.J. (2006). Evaluation of outcomes with citalopram for depression using measurement-based care in STAR*D: Implications for clinical practice. Am. J. Psychiatry.

[31] Sharifi-Rad J., Quispe C., Herrera-Bravo J., Martorell M., Sharopov F., Tumer T.B., Kurt B., Lankatillake C., Docea A.O., Moreira A.C. (2021). A Pharmacological Perspective on Plant-derived Bioactive Molecules for Epilepsy. Neurochem. Res..

[32] Nussbaum L., Hogea L.M., Calina D., Andreescu N., Gradinaru R., Stefanescu R., Puiu M. (2017). Modern treatment approaches in psychoses. pharmacogenetic, neuroimagistic and clinical implications. Farmacia.

[33] Sanacora G., Treccani G., Popoli M. (2012). Towards a glutamate hypothesis of depression: An emerging frontier of neuropsychopharmacology for mood disorders. Neuropharmacology.

[34] Douen A.G., Akiyama K., Hogan M.J., Wang F., Dong L., Chow A.K., Hakim A. (2000). Preconditioning with cortical spreading depression decreases intraischemic cerebral glutamate levels and down-regulates excitatory amino acid transporters EAAT1 and EAAT2 from rat cerebal cortex plasma membranes. J. Neurochem..

[35] Lin C.I., Orlov I., Ruggiero A.M., Dykes-Hoberg M., Lee A., Jackson M., Rothstein J.D. (2001). Modulation of the neuronal glutamate transporter EAAC1 by the interacting protein GTRAP3-18. Nature.

[36] Islam M.S., Quispe C., Hossain R., Islam M.T., Al-Harrasi A., Al-Rawahi A., Martorell M., Mamurova A., Seilkhan A., Altybaeva N. (2021). Neuropharmacological Effects of Quercetin: A Literature-Based Review. Front. Pharmacol..

[37] Calina D., Buga A.M., Mitroi M., Buha A., Caruntu C., Scheau C., Bouyahya A., El Omari N., El Menyiy N., Docea A.O. (2020). The Treatment of Cognitive, Behavioural and Motor Impairments from Brain Injury and Neurodegenerative Diseases through Cannabinoid System Modulation-Evidence from In Vivo Studies. J. Clin. Med..

[38] Pan L.L., Qi R.R., Wang J.Q., Zhou W., Liu J.L., Cai Y.L. (2016). Evidence for a Role of Orexin/Hypocretin System in Vestibular Lesion-Induced Locomotor Abnormalities in Rats. Front. Neurosci.-Switz.

[39] Henningsen K., Palmfeldt J., Christiansen S., Baiges I., Bak S., Jensen O.N., Gregersen N., Wiborg O. (2012). Candidate hippocampal biomarkers of susceptibility and resilience to stress in a rat model of depression. Mol. Cell Proteom..

[40] Chandley M.J., Szebeni K., Szebeni A., Crawford J., Stockmeier C.A., Turecki G., Miguel-Hidalgo J.J., Ordway G.A. (2013). Gene expression deficits in pontine locus coeruleus astrocytes in men with major depressive disorder. J. Psychiatr. Neurosci..

[41] Liu Y., Ding X.F., Wang X.X., Zou X.J., Li X.J., Liu Y.E.Y., Li J., Qian X.Y., Chen J.X. (2019). Xiaoyaosan exerts antidepressant-like effects by regulating the functions of astrocytes and EAATs in the prefrontal cortex of mice. BMC Complem. Altern. Med..

[42] Sun H., Su R., Zhang X., Wen J., Yao D., Gao X., Zhu Z., Li H. (2017). Hippocampal GR- and CB1-mediated mGluR5 differentially produces susceptibility and resilience to acute and chronic mild stress in rats. Neuroscience.

[43] Maier S., Reiterer V., Ruggiero A.M., Rothstein J.D., Thomas S., Dahm R., Sitte H.H., Farhan H. (2009). GTRAP3-18 serves as a negative regulator of Rab1 in protein transport and neuronal differentiation. J. Cell Mol. Med..

[44] Wang R., Zhao X., Xu J., Wen Y., Li A., Lu M., Zhou J. (2018). Astrocytic JWA deletion exacerbates dopaminergic neurodegeneration by decreasing glutamate transporters in mice. Cell Death Dis..

[45] Altar C.A., Vawter M.P., Ginsberg S.D. (2009). Target Identification for CNS Diseases by Transcriptional Profiling. Neuropsychopharmacology.

[46] Aloizou A.M., Siokas V., Pateraki G., Liampas I., Bakirtzis C., Tsouris Z., Lazopoulos G., Calina D., Docea A.O., Tsatsakis A. (2021). Thinking Outside the Ischemia Box: Advancements in the Use of Multiple Sclerosis Drugs in Ischemic Stroke. J. Clin. Med..

[47] Pereira C.M., Oliveira C.R. (1997). Glutamate toxicity on a PC12 cell line involves glutathione (GSH) depletion and oxidative stress. Free Radic. Biol. Med..

[48] Shaw C.A., Bains J.S. (2002). Synergistic versus antagonistic actions of glutamate and glutathione: The role of excitotoxicity and oxidative stress in neuronal disease. Cell Mol. Biol..

[49] Tsatsakis A., Docea A.O., Calina D., Tsarouhas K., Zamfira L.-M., Mitrut R., Sharifi-Rad J., Kovatsi L., Siokas V., Dardiotis E. (2019). A Mechanistic and Pathophysiological Approach for Stroke Associated with Drugs of Abuse. J. Clin. Med..

[50] Pessoa L. (2008). On the relationship between emotion and cognition. Nat. Rev. Neurosci..

[51] Cai W., Ma W., Wang G.T., Li Y.J., Shen W.D. (2019). Antidepressant, anti-inflammatory, and antioxidant effects of electroacupuncture through sonic hedgehog-signaling pathway in a rat model of poststroke depression. Neuropsychiatr. Dis. Treat..

[52] Mineur Y.S., Obayemi A., Wigestrand M.B., Fote G.M., Calarco C.A., Li A.M., Picciotto M.R. (2013). Cholinergic signaling in the hippocampus regulates social stress resilience and anxiety- and depression-like behavior. Proc. Natl. Acad. Sci. USA.

[53] Lau C.G., Takeuchi K., Rodenas-Ruano A., Takayasu Y., Murphy J., Bennett M.V.L., Zukin R.S. (2009). Regulation of NMDA receptor Ca^2+^ signalling and synaptic plasticity. Biochem. Soc. Trans..

[54] Khadrawy Y.A., Hosny E.N., Magdy M., Mohammed H.S. (2021). Antidepressant effects of curcumin-coated iron oxide nanoparticles in a rat model of depression. Eur. J. Pharmacol..

[55] Dong H., Xiang Y.Y., Farchi N., Ju W., Wu Y., Chen L., Wang Y., Hochner B., Yang B., Soreq H. (2004). Excessive expression of acetylcholinesterase impairs glutamatergic synaptogenesis in hippocampal neurons. J. Neurosci..

[56] Dilsaver S.C., Peck J.A., Overstreet D.H. (1992). The Flinders Sensitive Line exhibits enhanced thermic responsiveness to nicotine relative to the Sprague-Dawley rat. Pharmacol. Biochem. Behav..

[57] Lai T.W., Zhang S., Wang Y.T. (2014). Excitotoxicity and stroke: Identifying novel targets for neuroprotection. Prog. NeuroBiol..

[58] Salehi B., Sestito S., Rapposelli S., Peron G., Calina D., Sharifi-Rad M., Sharopov F., Martins N., Sharifi-Rad J. (2019). Epibatidine: A Promising Natural Alkaloid in Health. Biomolecules.

[59] Buga A.M., Docea A.O., Albu C., Malin R.D., Branisteanu D.E., Ianosi G., Ianosi S.L., Iordache A., Calina D. (2019). Molecular and cellular stratagem of brain metastases associated with melanoma. Oncol. Lett..

[60] Li L.L., Ginet V., Liu X.N., Vergun O., Tuittila M., Mathieu M., Bonny C., Puyal J., Truttmann A.C., Courtney M.J. (2013). The nNOS-p38MAPK Pathway Is Mediated by NOS1AP during Neuronal Death. J. Neurosci..

[61] Ehsanifar M., Tameh A.A., Farzadkia M., Kalantari R.R., Zavareh M.S., Nikzaad H., Jafari A.J. (2019). Exposure to nanoscale diesel exhaust particles: Oxidative stress, neuroinflammation, anxiety and depression on adult male mice. Ecotoxicol. Environ. Saf..

[62] Salehi B., Sharifi-Rad J., Cappellini F., Reiner A., Zorzan D., Imran M., Sener B., Kilic M., El-Shazly M., Fahmy N.M. (2020). The Therapeutic Potential of Anthocyanins: Current Approaches Based on Their Molecular Mechanism of Action. Front. Pharmacol..

[63] Ng F., Berk M., Dean O., Bush A.I. (2008). Oxidative stress in psychiatric disorders: Evidence base and therapeutic implications. Int. J. Neuropsychopharmacol..

[64] Black C.N., Bot M., Scheffer P.G., Cuijpers P., Penninx B.W. (2015). Is depression associated with increased oxidative stress? A systematic review and meta-analysis. Psychoneuroendocrinology.

[65] Orlovsky M.A., Dosenko V.E., Spiga F., Skibo G.G., Lightman S.L. (2014). Hippocampus remodeling by chronic stress accompanied by GR, proteasome and caspase-3 overexpression. Brain Res..

[66] Santha P., Veszelka S., Hoyk Z., Meszaros M., Walter F.R., Toth A.E., Kiss L., Kincses A., Olah Z., Seprenyi G. (2016). Restraint Stress-Induced Morphological Changes at the Blood-Brain Barrier in Adult Rats. Front. Mol. Neurosci..

